# TRIM21: a multifaceted regulator in cancer

**DOI:** 10.3389/fcell.2025.1637451

**Published:** 2025-07-15

**Authors:** Aizhuo Li, Jiannan Wang, Yi Qu

**Affiliations:** ^1^ Department of Ultrasound, The First Affiliated Hospital of China Medical University, Shenyang, China; ^2^ Department of Hematology, The First Affiliated Hospital of China Medical University, Shenyang, China

**Keywords:** cancer, ubiquination, metabolism, autophagy, immunity

## Abstract

Ubiquitination serves as a dynamic post-translational modification that enables rapid and precise regulation of cellular signaling networks. TRIM21, as an important member of the TRIM family, is a protein with E3 ubiquitin ligase activity. By specifically recognizing and ubiquitinating various substrate proteins, it plays a pivotal regulatory role in tumorigenesis and development. Moreover, TRIM21 has been found to play a multi-faceted role in cellular autophagy, metabolic reprogramming, immune escape, tumor proliferation, metastasis and resistance to cell death by regulating the stability and function of key proteins. In this review, we provided an in-depth understanding of the specific mechanism of TRIM21 in different biological processes and tumor types, which contributes to the development of novel targeted therapeutic strategies targeting TRIM21.

## Introduction

**FIGURE 1:**
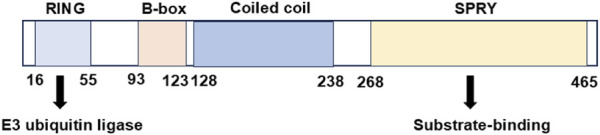
The domains of tripartite motif 21 (TRIM21) protein.

The ubiquitin-proteasome system (UPS) constitutes a tightly regulated mechanism for protein degradation, playing a pivotal role in sustaining cellular homeostasis. The dysregulation of ubiquitin-mediated proteasomal degradation has emerged as a hallmark for tumors (Cockram et al., 2021). The tripartite motif (TRIM) protein family represents one of the largest classes of single protein RING finger E3 ubiquitin ligases, containing more than 80 members with diverse cellular roles in intracellular signaling, immune response, autophagy, and tumorigenesis (Hatakeyama, 2017; Liu et al., 2020; Huang et al., 2022). TRIM21, initially discovered as an antibody-binding protein in autoimmune diseases, exhibits a multidomain architecture that underpins its function. TRIM21 possesses an N-terminal RING domain with E3 ubiquitin ligase activity, a B-box domain, a coiled-coil domain, and a C-terminal substrate-binding domain (PRY/SPRY) (Figure 1). It has been found that TRIM21 participates in the regulation of biological processes, such as autophagy, signal transduction, immune response and tumorigenesis. Moreover, TRIM21 exhibits a context-dependent dual role in cancer progression (Table 1). It can act as both a tumor suppressor or a pro-tumor driver according to diverse cellular microenvironment. Here, we will review the biological role of TRIM21 in human malignancies and discuss possible therapeutic interventions targeting TRIM21 for cancer treatment.

**TABLE 1:** The dual role of TRIM21 in different cancer types.

Cancer type	Phenotypic effects	References
Pro-tumor effects of TRIM21		
Glioblastoma	TRIM21 overexpression promotes invasion and growth of glioblastoma	Li et al. (2023a)
Gastrointestinal stromal tumors	TRIM21 overexpression promotes imatinib resistance	Cui et al. (2024)
Nasopharyngeal carcinoma	TRIM21 knockout activates cytotoxic T cell-mediated anti-tumor immunity in response to radiation	Li et al. (2023b)
Pancreatic ductal adenocarcinoma	TRIM21 knockout sensitizes tumor cells to ferroptosis	Gong et al. (2023)
Pancreatic Cancer	TRIM21 knockout inhibits tumor proliferation and gemcitabine resistance	Fan et al. (2025)
Anti-tumor effects of TRIM21		
Breast cancer and colorectal caner	TRIM21 overexpression inhibits anchorage-independent growth of SK-BR3 and HT29; TRIM21 knockout promotes the growth of SK-BR3 and HT29 *in vivo*	Liu et al. (2023a)
Renal cell carcinoma	TRIM21 overexpression inhibits tumor growth	Chen et al. (2023)
Triple-negative breast cancer	TRIM21 overexpression suppresses M2 macrophage polarization and tumor progression	Zhang et al. (2023)
Colorectal cancer	TRIM21 overexpression sensitizes regorafenib therapy in colorectal cancer	Ye et al. (2024)
T cell acute lymphoblastic leukemia and prostate cancer	TRIM21 knockout promotes cell proliferation	Cheng et al. (2020)

## Regulation of TRIM21 expression and function

TRIM21 expression has been found to be regulated by transcriptional mechanisms. For instance, TRIM21 expression is upregulated by stimulation with interferon (IFNs) induced by interferon regulatory factors (IRFs) (Yang et al., 2009). However, the detailed mechanism of TRIM21 transcription remains to be explored. Post-translational modification is essential for the stability and functions of TRIM21. The mutual regulation between TRIM family members has been reported. A direct mutual regulation between TRIM21 and TRIM8 has been found in lung and renal cancer cells, by activating their proteasome pathway via Lys48 (K48)- linked ubiquitination (Wang et al., 2023). UBE2M has been found to mediate the neddylation of TRIM21 and promote ubiquitination degradation of Von Hippel-Lindau (VHL) tumor suppressor by increasing TRIM21 and VHL interactions (Lu et al., 2023). TRIM21 is oxidized at C92, C111, and C114 to form disulfide bonds that lead to its oligomerization and decreased E3 activity (Yang et al., 2025).

## Biological roles of TRIM21 in human malignancies

Accumulating studies have shown that TRIM21 positively and negatively regulate carcinogenesis in different context of cancers. It has been reported that TRIM21 participates in sustaining cell proliferation, autophagy, tumor proliferation, metastasis and anti-tumor immunity. In this section, we primarily discussed the regulatory role of TRIM21 on these different biological processes in human malignancies.

### Regulating cellular autophagy

Autophagy is an evolutionarily conserved self-degradation process essential for cellular homeostasis under stress (Mizushima and Komatsu, 2011). This pathway relies on ATG proteins and core complexes including the ULK1 initiation complex, PI3K-Atg14 complex, ATG9A vesicle delivery system, and ATG12/LC3 conjugation systems, which collectively regulate autophagosome formation, maturation and degradation (Miller and Thorburn, 2021). Recent reports have shown that TRIM21 interacts with multiple regulators and receptors of autophagy, including ULK1, BECN1 and SQSTM1/p62, therefore regulating cellular autophagy (Yang et al., 2025; Zhao L. et al., 2022). In gastric cancer stem cells, TRIM21 mediates the degradation of key autophagy protein ULK1 and induced the K63-mediated ubiquitination of ULK1to activate autophagy (Lin and Hurley, 2016; Zhao R. et al., 2022).

**FIGURE 2:**
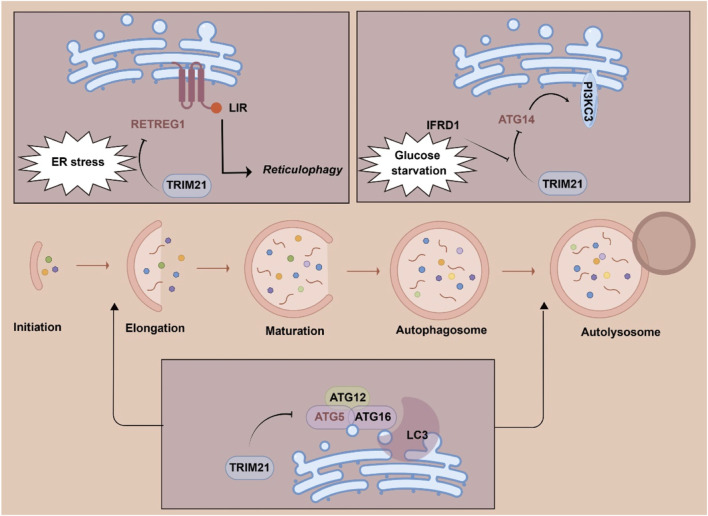
Substrates of tripartite motif 21 (TRIM21) in cellular autophagy. Abbreviations: ER, endoplasmic reticulum; IFRD1, interferon-related developmental regulator 1; LIR, LC3-interacting region; RETREG1, reticulophagy regulator 1.

TRIM21 has been reported to directly interact with multiple autophagy machinery proteins and mediate the proteasomal degradation of these proteins (Figure 2) (Kimura et al., 2017; Zhao L. et al., 2022; Huang et al., 2024). ATG5 is a key player of the ATG12-ATG5 conjugation system that promotes LC3 conjugation to the autophagic membrane (Corkery et al., 2023). TRIM21 has been found to target ATG5 and mediate K48-linked ATG5 ubiquitination and degradation to block pro-survival autophagy in multiple myeloma cells. ATG14, an essential regulator for the fusion of autophagosomes with lysosomes, has also been identified as the substrate of TRIM21. Under glutamine starvation, TRIM21 mediates the proteasomal degradation of the key autophagy regulator ATG14 to inhibit autophagy (Huang et al., 2024). In hepatocellular carcinoma (HCC), interferon-related developmental regulator 1 (IFRD1) is upregulated by glutamine starvation to inhibit autophagy by promoting TRIM21-mediated degradation ATG14 (Huang et al., 2024). Reticulophagy regulator 1 (RETREG1) is a well-characterized endoplasmic reticulum (ER) autophagy (reticulophagy) receptor (Zhang et al., 2024). It has been found that TRIM21 ubiquitinates RETREG1 at K247 and K252 to promote its proteasomal degradation. Conversely, cytoskeleton-associated protein 4 (CKAP4) competes with TRIM21 to bind RETREG1, which protects RETREG1 from degradation. In hepatocellular carcinoma, stress-induced TRIM21 upregulation mitigates the function of RETREG1 to restore ER stress equilibrium (Mo et al., 2025). Collectively, TRIM21-mediated ubiquitination of autophagy machinery proteins is critical for regulating cellular autophagy.

### Reprogramming cellular metabolism

**FIGURE 3:**
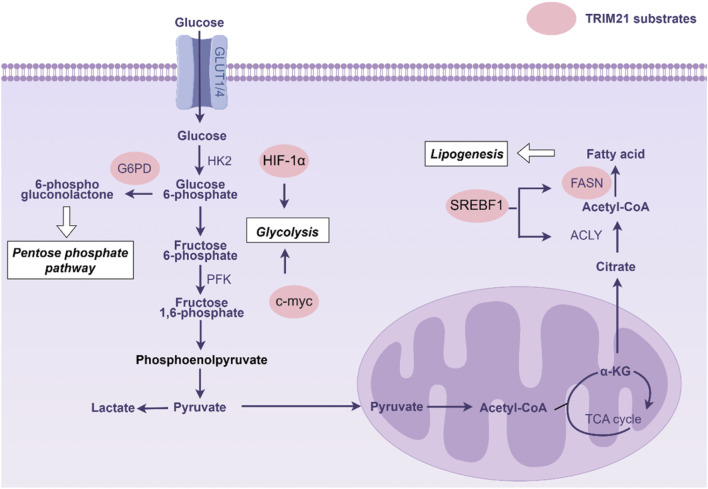
Substrates of tripartite motif 21 (TRIM21) in cellular metabolism. Abbreviations: ACLY, ATP citrate lyase; FASN, Fatty acid synthase; HK2, Hexokinase 2; G6PD, Glucose-6-phosphate dehydrogenase; PFK, Phosphofructokinase; SREBF-1, Sterol regulatory element-binding protein gene.

Metabolic adaptation is an emerging hallmark of tumors. TRIM21-mediated ubiquitination of key metabolic enzymes or transcriptional regulator is involved in the regulation of cellular metabolism (Figure 3). Through glycolysis, tumor cells convert glucose into lactate and rapidly produce energy to support tumor proliferation (Paul et al., 2022). Hypoxia-inducible factor-1 alpha (HIF-1α) serves as a master transcriptional regulator of glycolytic genes (Semenza, 2003). In renal cell carcinoma, TRIM21 targets HIF-1α for ubiquitin-mediated degradation, thereby suppressing HIF-1α-dependent glycolytic programming in tumor cells (Chen et al., 2021). This regulatory axis demonstrates the critical role of TRIM21 in modulating cancer metabolism through post-translational control of metabolic transcription factors. c-Myc serves as a master regulator of aerobic glycolysis through direct transcriptional activation of glycolytic enzymes (Fang et al., 2019). Recent studies reveal that TRIM21 modulates this metabolic pathway by specifically recognizing c-Myc and catalyzing K63-linked ubiquitination at lysine 148. This post-translational modification targets c-Myc for autophagic degradation, leading to suppressed enolase 2 expression and consequent glycolysis inhibition (Ye et al., 2024).

The glycolytic intermediates can be diverted into the pentose phosphate pathway (PPP) to fuel nucleotide biosynthesis and generate NADPH, supporting cell proliferation and redox homeostasis (TeSlaa et al., 2023). TRIM21 critically regulates this metabolic branch by targeting glucose-6-phosphate dehydrogenase (G6PD), the rate-limiting enzyme of the oxidative PPP, for ubiquitin-dependent degradation. Mass spectrometry identified eight lysine residues on G6PD as ubiquitination sites, and mutation of these sites significantly stabilized G6PD (Cheng et al., 2020). Notably, oncogenic PI3K/AKT activation or PTEN loss suppressed TRIM21 expression, thereby elevating G6PD activity and PPP flux. Metabolites derived from PPP further potentiate AKT signaling and amplify tumorigenic metabolic reprogramming (Cheng et al., 2020).

Lipid metabolism fuels tumor growth by providing essential biomolecules and energy (Currie et al., 2013). FASN, the key enzyme *de novo* lipogenesis, is frequently overexpressed in cancers. FASN inhibition disrupts membrane synthesis, survival signaling, and energy homeostasis, making it a promising metabolic target for cancer treatment (Menendez and Lupu, 2007). FASN has been identified as a key substrate of TRIM21 (Gu et al., 2020). The nuclear neddylated PTEN dephosphorylates FASN to reduce the TRIM21-mediated ubiquitylation and degradation of FASN, and then promotes *de novo* fatty acid synthesis. ACAT1 acetylates GNPAT at K128, which represses TRIM21-mediated GNPAT ubiquitination and degradation. GNPAT represses TRIM21-mediated FASN degradation and promotes lipid metabolism and hepatocarcinogenesis. However, FASN acetylation enhanced its association with the E3 ubiquitin ligase TRIM21. Acetylation destabilized FASN and resulted in decreased *de novo* lipogenesis and tumor cell growth (Gu et al., 2020). In renal cell carcinoma, TRIM21 mediates ubiquitination-mediated degradation of sterol regulatory element binding transcription factor 1 (SREBF1) to inhibit the expression of lipogenic enzymes, thereby blocking lipogenesis and tumor development (Chen et al., 2023). Microsomal epoxide hydrolase 1 (EPHX1) metabolizes 2-arachidonoylglycerol (2-AG) into arachidonic acid (AA) and glycerol, and has been identified as a direct target of TRIM21 in pancreatic cancer. TRIM21 binds EPHX1 via its SPRY domain and induces proteasomal degradation through K33- and K48-linked ubiquitination at lysine 105 (K105). Functionally, EPHX1-derived AA promotes ferroptosis and suppresses tumor proliferation, whereas TRIM21-mediated EPHX1 degradation sustains AA depletion, thereby enhancing pancreatic cancer growth and conferring gemcitabine resistance (Fan et al., 2025). Collectively, the specific roles of TRIM21 in modulating tumor metabolism, especially lipid metabolism, and its interaction with metabolic networks require further exploration.

### Regulating immune evasion

Immune evasion is an emerging hallmark contributing to tumor development (Galassi et al., 2024). Tumor cells employ a variety of immunosuppressive mechanisms to escape from immune surveillance by regulating the function of CD8^+^ T cell, tumor-associated macrophages and other components in the TME (Vinay et al., 2015). In this section, we focused on the role of TRIM21 in the regulation of immune evasion in different context of cancers.

TRIM21 has also been found to impair CD8^+^ T cell activation and anti-tumor immunity in some contexts of cancers. TRIM21 could catalyze the K63-linked polyubiquitination on programmed cell death-1 (PD-1) at K233, resulting in stabilization of PD-1 by antagonizing its K48-linked polyubiquitination and degradation to impair anti-tumor immunity of CD8^+^T cells (Shi et al., 2025). Conversely, TRIM21 deficiency significantly decreased PD-1 expression and activated cytotoxic CD8^+^ T cells, which sensitizes tumors to anti-CTLA-4 immunotherapy. In hepatocellular carcinoma (HCC), ependymin-related protein 1 (EPDR1) plays a regulatory role on PD-L1 expression to mediate immune evasion in a TRIM21-dependent manner. Mechanistically, EPDR1 binds to TRIM21 and reduce TRIM21-dependent degradation of IkappaB kinase-b to promote NF-κB-mediated transcriptional activation of PD-L1 (Qian et al., 2024).

To achieve immune evasion, tumor cells alter their expression of E3 ubiquitin ligases that regulate the function of CD8^+^ T cell and anti-tumor immunity. Among these E3 ligases, TRIM21 enhances the cytotoxic function of CD8^+^ T cell function within TME by promoting the K48-linked ubiquitination and degradation of the mitochondrial voltage-dependent anion-selective channel protein 2 (VDAC2). TRIM21-mediated VDAC2 degradation inhibits pore formation by VDAC2 oligomers for cytosolic mtDNA release, thus suppressing radiation-induced STING–type-I IFN signalling. Conversely, TRIM21 deficiency enhances VDAC2 oligomerization-mediated cytosolic mtDNA release, activates the cGAS/STING cytosolic DNA sensing pathway, potentiates the antigen-presenting capacity of tumor cells, and activates cytotoxic T cell-mediated anti-tumor immunity in response to radiation (Li JY. et al., 2023). Extracellular adenosine has been found at high levels in the TME, which creates an immunosuppressive microenvironment suppressing the anti-tumor effects of cytotoxic T cells (Zahavi and Hodge, 2023). TRIM21 functions as an E3 ligase that mediates the ubiquitin-proteasomal degradation of CD73, a pivotal enzyme mediating the conversion of ATP to adenosine (Fu et al., 2023). In triple-negative breast cancers (TNBCs), TRIM21 knockdown could stabilize CD73 protein and promote CD73-mediated adenosine accumulation, dampening CD8^+^ T cell function. Findings from a recent study showed that deubiquitylation of CD73 by OTUD4 counteracted its ubiquitylation by TRIM21. While TRIM21 promotes the degradation of CD73 protein, deubiquitination of CD73 by OTUD4 stabilizes CD73 and impair CD8^+^ T cell function via adenosine production (Zhu et al., 2024). Given this immune suppressive role of CD73, ST80 blocks the interaction between OTUD4 and CD73, to promote CD73 proteolysis and restore capacity to elicit anti-tumor responses of CD8^+^ T cell in immune-suppressive TNBCs.

Anti-phagocytic signals to avoid self-elimination by phagocytes are critical for governing the evasion of tumor cells from immune surveillance (Liu et al., 2017). CD47 is a tumor-associated antigen binds to and activates signal regulatory protein α (SIRPα), an inhibitory protein expressed on the surface of macrophages, allowing tumor cells to evade innate immune surveillance. TRIM21 has been identified as the E3 ligase that mediates the polyubiquitylation and degradation of CD47 at lysine 99 and 102. However, c-Src-mediated CD47 phosphorylation inhibits the interaction between TRIM21 and CD47, thereby abrogating TRIM21-mediated CD47 polyubiquitylation and degradation to promote immune evasion (Du et al., 2023). CD4^+^ T cells, a type of inflammatory cell, are crucial in supporting and sustaining antitumor immune responses, which are predominantly mediated by the release of cytokines into the tumor microenvironment. Activated CD4^+^ T cells exhibit high expression levels of CD40L, which in turn enhances the antitumor immune response. In breast cancer, TRIM21 mediated the ubiquitination and degradation of CCT2 to promotes CD4+T cell activation. impair the pro-tumor effects of CCT2. Mechanistically, exosomal CCT2 inhibited Ca^2+^-NFAT1 signaling, thereby reducing CD40L expression on CD4^+^T cell (Chen et al., 2024).

### Sustaining cell proliferation

Sustained cell proliferation can be explained by mutations in oncogenes and tumor suppressors that regulate cell growth. The p53 tumor suppressor plays a key role in the maintenance of the genome integrity by inhibiting the proliferation of cells with damaged DNA (Blandino et al., 2020). Mutations in the p53 gene have been correlated with multiple types of tumors. Moreover, the proteasomal degradation of p53 tumor suppressor is regulated by polyubiquitination. TRIM21 has been found to promote tumorigenesis by destabilizing p53 (Hock AK and Vousden, 2014). However, TRIM21 directly interacted with mutant p53 but not wildtype p53, thereby leading to ubiquitination and degradation of mutant p53 to impair the role of mutant p53 “gain of function” in tumorigenesis. Conversely, TRIM21 deletion led to mutant p53 accumulation and gain of function to impair tumorigenesis (Liu J. et al., 2023).

The activation of proliferative signaling is also indispensable for sustaining cell proliferation. The canonical Wnt signaling pathway is closely implicated in tumorigenesis (Latour et al., 2021). Once bound by Wnt, Frizzled/LDL-recepor-related protein promotes translocation of β-catenin from the cytoplasm to the nucleus to activate T-cell factor (TCF)/lymphoid enhancer binding factor (LEF) and drive the upregulation of Wnt target genes for tumor proliferation (MacDonald et al., 2009). TRIM21 not only promotes K63-linked ubiquitination of β-catenin, accelerating its translocation into nuclei, but also increases β-catenin in nucleus by enhancing K48-linked ubiquitination of TIF1γ, another important regulator of β-catenin. TRIM21 forms a complex with the β-catenin upstream regulator, TIF1γ, in the nucleus and accelerated its degradation by inducing K48-linked ubiquitination at K5 site, to increase the level of nuclear β-catenin for tumor proliferation (Li Y. et al., 2023). Therefore, targeting TRIM21 is a promising therapeutic strategy for glioma with hyperactive β-catenin.

### Regulating tumor metastasis

TRIM21-mediated ubiquitination is critical for protein stability, activity, modification, or cellular localization of substrate protein, therefore participating in the regulation of tumor metastasis. For instance, tyrosine aminotransferase (TAT) is a key regulator for liver metastasis of gallbladder cancer by potentiating cardiolipin-dependent mitophagy. Moreover, TRIM21 mediated the K63-linked ubiquitination on TAT at K136 to impair its dimerization and mitochondrial location, subsequently inhibiting tumor invasion and migration of gallbladder cells. Given that TAT as a pro-metastasis regulator, TRIM21 may exert inhibitory effects on liver metastasis of gallbladder cancer. However, Xiao et al. found that UBE2S could interact with TRIM21 and coordinately mediated the ubiquitination of lipoma preferred partner (LPP) via K11-linked polyubiquitination to promote the lymphatic metastasis of gallbladder cancer (Xiao et al., 2023). Hippo and its downstream effectors, the transcriptional co-activators Yes-associated protein (YAP) are critical transcriptional regulators involved in human malignancies (Zanconato et al., 2016). The Hippo signaling is regulated by a series of kinases, including MST1/2. TRIM21 functions as an inhibitory regulator on the metastatic potential of colorectal cancer by regulating Hippo/YAP signaling pathway. Mechanistically, TRIM21 mediated the K63-linked polyubiquitination of MST2 at lysine 473, leading to the formation of MST2 homodimer and increased kinase activity for the functional inactivation of YAP (Liu YX. et al., 2023). In addition, TRIM21 binds to PRMT1 via its SPRY domain to promote the ubiquitination and degradation of the oncogene PRMT1 in a K48-linked manner, thereby inhibiting the metastasis of colorectal cancer cells (Cao et al., 2025).

### Resisting cell death

Ferroptosis is an iron-dependent regulated cell death characterized by excessive lipid peroxide accumulation. TRIM21 acts as a ferroptosis gatekeeper by targeting key proteins involved in ferroptosis. For instance, TRIM21 catalyzes K48-linked polyubiquitination and degradation of acyl-CoA synthetase long-chain family member 4 (ACSL4), a critical enzyme for incorporating polyunsaturated fatty acids (PUFAs) into phospholipids that dictates ferroptosis sensitivity, to promote ferroptosis resistance (Cui et al., 2024). In addition, TRIM21 binds ferroptosis suppressor protein 1 (FSP1) and mediates K63-linked ubiquitination at residues K322/K366. This modification promotes FSP1 membrane localization which is essential for its membrane translocation and ferroptosis suppression ability (Gong et al., 2023).

## TRIM21 as a prognostic marker of human malignancies

It has been found that the expression of TRIM21 is dysregulated in a broad spectrum of malignancies. Both aberrant upregulation and downregulation of TRIM21 in tumor tissues has been found to be correlated with adverse clinicopathological characteristics and poor prognosis, suggesting its potential as a context-dependent prognostic biomarker. Therefore, it is essential to characterize the clinicopathological parameters affected by TRIM21 expression for guiding optimal therapeutic strategies in clinical practice. In 355 patients with nasopharyngeal carcinoma, high TRIM21 expression was correlated with shorter locoregional recurrence-free survival, disease-free survival, and overall survival times (Li JY. et al., 2023). In 120 glioma samples, high TRIM21 protein level was correlated with advanced tumor stage. Multivariate analyses revealed that TRIM21 was an independent indicator for overall survival of patients with glioma (Li Y. et al., 2023). In pancreatic ductal adenocarcinoma, TRIM21 was upregulated in PDAC samples compared with non-tumor samples. Kaplan–Meier survival analysis revealed that high TRIM21 protein level was negatively correlated with the overall survival of PDAC patients (Gong et al., 2023).

However, poor clinical outcomes have been observed in tumors with decreased levels of CTGF expression relative to normal tissues. In renal cell carcinoma, low TRIM21 expression was significantly positively correlated with tumor size, lymph node metastasis, and distant metastasis (Chen et al., 2023). In colorectal and breast cancers, TRIM21 protein levels were frequently downregulated in colorectal and breast cancers. Moreover, low TRIM21 expression was significantly correlated with poor clinical outcomes in patients with mutp53 cancers but not wtp53 tumors (Liu J. et al., 2023).

## Clinical implications and future directions

Proteolysis-targeting chimera (PROTAC) has emerged as a promising technology for degradation of specific disease-related proteins (Zhao R. et al., 2022). Targeted protein degradation utilizes PROTACs to degrade cancer-related proteins by enhancing their bindings to E3 ubiquitin ligases for cancer treatment (Li and Song, 2020). Lu et al. developed a TRIM21-based PROTAC by functionalization of acepromazine, an anti-psychotic drug, into PROTACs to increase the interaction of TRIM21 with nucleoporin NUP98. Considering that aberrant protein assemblies are related to tumorigenesis, this TRIM21-based degraders promotes the degradation of nuclear pore proteins to impair nucleocytoplasmic trafficking (Lu et al., 2024). Fletcher et al. developed a TRIM21-based PROTAC to degrade Human antigen R (HuR), an RNA regulator, to inhibit tumor growth in pre-clinical models by regulating HuR-related biological process. A single domain antibody (VHH) was found to bind with HuR to inhibit HuR binding to RNA to impair tumor growth *in vivo*. The HuR-targeting TRIM21-based PROTAC has been exploited as a promising strategy for regulating HuR expression and exerting anti-tumorigenic effects in pre-clinical models (Fletcher et al., 2023).

As we have discussed in this review, TRIM21 have many functions in a broad range of biological processes. Recent studies have established TRIM21 knockout mice and found TRIM21 as a pivotal regulator of tumorigenesis *in vivo* by a genetic approach using transgenic or knockout mice. Notably, it is essential to decipher the interacting proteins or substrates of TRIM21 to determine the multi-functionality of TRIM21. Further studies are required to fully characterize the complex regulation of TRIM21 in different cancer contexts and to dissect its underlying mechanisms. Besides, the high-specificity targeting properties of TRIM21-based therapeutic strategy are especially important. Thus, future perspectives include the development of TRIM21-based therapeutic strategy and their effective delivery into cancer cells.

## Conclusion

Ubiquitination serves as a dynamic post-translational modification that enables rapid and precise regulation of cellular signaling networks. TRIM21, as an E3 ubiquitin ligase, plays a multi-faceted role in tumorigenesis, metabolic reprogramming, immune escape by regulating the stability and function of key proteins. The diversity of its substrate selection and the specificity of the ubiquitin chain enable it to precisely regulate carcinogenic or tumor suppressor pathways. The complex regulatory network of TRIM21 suggests its great potential as a tumor therapeutic target, but its “double-edged sword” characteristics need to be overcome in the future.

## References

[B1] Blandino G. Valenti F. Sacconi A. Di Agostino S. (2020). Wild type- and mutant p53 proteins in mitochondrial dysfunction: emerging insights in cancer disease. Semin. Cell Dev. Biol. 98, 105–117. 10.1016/j.semcdb.2019.05.011 31112799

[B2] Cao M. Shao Z. Qian X. Chen M. Deng C. Chen X. (2025). TRIM21-mediated PRMT1 degradation attenuates colorectal cancer malignant progression. Cell Death Dis. 16 (1), 56. 10.1038/s41419-025-07383-9 39890802 PMC11785787

[B3] Chen X. Li Z. Yong H. Wang W. Wang D. Chu S. (2021). Trim21-mediated HIF-1α degradation attenuates aerobic glycolysis to inhibit renal cancer tumorigenesis and metastasis. Cancer Lett. 508, 115–126. 10.1016/j.canlet.2021.03.023 33794309

[B4] Chen X. Ma C. Li Y. Liang Y. Chen T. Han D. (2024). Trim21-mediated CCT2 ubiquitination suppresses malignant progression and promotes CD4(+)T cell activation in breast cancer. Cell Death Dis. 15 (7), 542. 10.1038/s41419-024-06944-8 39079960 PMC11289294

[B5] Chen X. Yong H. Chen M. Deng C. Wang P. Chu S. (2023). TRIM21 attenuates renal carcinoma lipogenesis and malignancy by regulating SREBF1 protein stability. J. Exp. Clin. Cancer Res. 42 (1), 34. 10.1186/s13046-022-02583-z 36694250 PMC9875457

[B6] Cheng J. Huang Y. Zhang X. Yu Y. Wu S. Jiao J. (2020). TRIM21 and PHLDA3 negatively regulate the crosstalk between the PI3K/AKT pathway and PPP metabolism. Nat. Commun. 11 (1), 1880. 10.1038/s41467-020-15819-3 32312982 PMC7170963

[B7] Cockram P. E. Kist M. Prakash S. Chen S. H. Wertz I. E. Vucic D. (2021). Ubiquitination in the regulation of inflammatory cell death and cancer. Cell Death Differ. 28 (2), 591–605. 10.1038/s41418-020-00708-5 33432113 PMC7798376

[B8] Corkery D. P. Castro-Gonzalez S. Knyazeva A. Herzog L. K. Wu Y. W. (2023). An ATG12-ATG5-TECPR1 E3-like complex regulates unconventional LC3 lipidation at damaged lysosomes. EMBO Rep. 24 (9), e56841. 10.15252/embr.202356841 37381828 PMC10481663

[B9] Cui Z. Sun H. Gao Z. Li C. Xiao T. Bian Y. (2024). TRIM21/USP15 balances ACSL4 stability and the imatinib resistance of gastrointestinal stromal tumors. Br. J. Cancer 130 (4), 526–541. 10.1038/s41416-023-02562-x 38182686 PMC10876985

[B10] Currie E. Schulze A. Zechner R. Walther T. C. Farese R. V. Jr (2013). Cellular fatty acid metabolism and cancer. Cell Metab. 18 (2), 153–161. 10.1016/j.cmet.2013.05.017 23791484 PMC3742569

[B11] Du L. Su Z. Wang S. Meng Y. Xiao F. Xu D. (2023). EGFR-induced and c-Src-Mediated CD47 phosphorylation inhibits TRIM21-Dependent polyubiquitylation and degradation of CD47 to promote tumor immune evasion. Adv. Sci. (Weinh) 10 (27), e2206380. 10.1002/advs.202206380 37541303 PMC10520678

[B12] Fan X. Dai Y. Mo C. Li H. Luan X. Wang B. (2025). TRIM21 promotes tumor growth and gemcitabine resistance in pancreatic cancer by inhibiting EPHX1-Mediated arachidonic acid metabolism. Adv. Sci. (Weinh) 12 (8), e2413674. 10.1002/advs.202413674 39739616 PMC11848624

[B13] Fang Y. Shen Z. Y. Zhan Y. Z. Feng X. C. Chen K. L. Li Y. S. (2019). CD36 inhibits beta-catenin/c-myc-mediated glycolysis through ubiquitination of GPC4 to repress colorectal tumorigenesis. Nat. Commun. 10 (1), 3981. 10.1038/s41467-019-11662-3 31484922 PMC6726635

[B14] Fletcher A. Clift D. de Vries E. Martinez Cuesta S. Malcolm T. Meghini F. (2023). A TRIM21-based bioPROTAC highlights the therapeutic benefit of HuR degradation. Nat. Commun. 14 (1), 7093. 10.1038/s41467-023-42546-2 37925433 PMC10625600

[B15] Fu Z. Chen S. Zhu Y. Zhang D. Xie P. Jiao Q. (2023). Proteolytic regulation of CD73 by TRIM21 orchestrates tumor immunogenicity. Sci. Adv. 9 (1), eadd6626. 10.1126/sciadv.add6626 36608132 PMC9821867

[B16] Galassi C. Chan T. A. Vitale I. Galluzzi L. (2024). The hallmarks of cancer immune evasion. Cancer Cell 42 (11), 1825–1863. 10.1016/j.ccell.2024.09.010 39393356

[B17] Gong J. Liu Y. Wang W. He R. Xia Q. Chen L. (2023). TRIM21-Promoted FSP1 plasma membrane translocation confers ferroptosis resistance in human cancers. Adv. Sci. (Weinh) 10 (29), e2302318. 10.1002/advs.202302318 37587773 PMC10582465

[B18] Gu L. Zhu Y. Lin X. Tan X. Lu B. Li Y. (2020). Stabilization of FASN by ACAT1-mediated GNPAT acetylation promotes lipid metabolism and hepatocarcinogenesis. Oncogene 39 (11), 2437–2449. 10.1038/s41388-020-1156-0 31974474

[B19] Hatakeyama S. (2017). TRIM family proteins: roles in autophagy, immunity, and carcinogenesis. Trends Biochem. Sci. 42 (4), 297–311. 10.1016/j.tibs.2017.01.002 28118948

[B20] Hock A. K. Vousden K. H. (2014). The role of ubiquitin modification in the regulation of p53. Biochim. Biophys. Acta 1843 (1), 137–149. 10.1016/j.bbamcr.2013.05.022 23742843

[B21] Huang N. Sun X. Li P. Liu X. Zhang X. Chen Q. (2022). TRIM family contribute to tumorigenesis, cancer development, and drug resistance. Exp. Hematol. Oncol. 11 (1), 75. 10.1186/s40164-022-00322-w 36261847 PMC9583506

[B22] Huang Y. Meng F. Zeng T. Thorne R. F. He L. Zha Q. (2024). IFRD1 promotes tumor cells “low-cost” survival under glutamine starvation *via* inhibiting histone H1.0 nucleophagy. Cell Discov. 10 (1), 57. 10.1038/s41421-024-00668-x 38802351 PMC11130292

[B23] Kimura T. Jain A. Choi S. W. Mandell M. A. Johansen T. Deretic V. (2017). TRIM-Directed selective autophagy regulates immune activation. Autophagy 13 (5), 989–990. 10.1080/15548627.2016.1154254 26983397 PMC5446080

[B24] Latour M. Her N. G. Kesari S. Nurmemmedov E. (2021). WNT signaling as a therapeutic target for glioblastoma. Int. J. Mol. Sci. 22 (16), 8428. 10.3390/ijms22168428 34445128 PMC8395085

[B25] Li J. Y. Zhao Y. Gong S. Wang M. M. Liu X. He Q. M. (2023a). TRIM21 inhibits irradiation-induced mitochondrial DNA release and impairs antitumour immunity in nasopharyngeal carcinoma tumour models. Nat. Commun. 14 (1), 865. 10.1038/s41467-023-36523-y 36797289 PMC9935546

[B26] Li X. Song Y. (2020). Proteolysis-targeting chimera (PROTAC) for targeted protein degradation and cancer therapy. J. Hematol. Oncol. 13 (1), 50. 10.1186/s13045-020-00885-3 32404196 PMC7218526

[B27] Li Y. Bao L. Zheng H. Geng M. Chen T. Dai X. (2023b). E3 ubiquitin ligase TRIM21 targets TIF1γ to regulate β-catenin signaling in glioblastoma. Theranostics 13 (14), 4919–4935. 10.7150/thno.85662 37771771 PMC10526654

[B28] Lin M. G. Hurley J. H. (2016). Structure and function of the ULK1 complex in autophagy. Curr. Opin. Cell Biol. 39, 61–68. 10.1016/j.ceb.2016.02.010 26921696 PMC4828305

[B29] Liu J. Zhang C. Xu D. Zhang T. Chang C. Y. Wang J. (2023a). The ubiquitin ligase TRIM21 regulates mutant p53 accumulation and gain of function in cancer. J. Clin. Invest 133 (6), e164354. 10.1172/JCI164354 36749630 PMC10014102

[B30] Liu X. Kwon H. Li Z. Fu Y. X. (2017). Is CD47 an innate immune checkpoint for tumor evasion? J. Hematol. Oncol. 10 (1), 12. 10.1186/s13045-016-0381-z 28077173 PMC5225552

[B31] Liu Y. Tao S. Liao L. Li Y. Li H. Li Z. (2020). TRIM25 promotes the cell survival and growth of hepatocellular carcinoma through targeting Keap1-Nrf2 pathway. Nat. Commun. 11 (1), 348. 10.1038/s41467-019-14190-2 31953436 PMC6969153

[B32] Liu Y. X. Wan S. Yang X. Q. Wang Y. Gan W. J. Ye W. L. (2023b). TRIM21 is a druggable target for the treatment of metastatic colorectal cancer through ubiquitination and activation of MST2. Cell Chem. Biol. 30 (7), 709–725.e6. 10.1016/j.chembiol.2023.05.009 37354905

[B33] Lu P. Cheng Y. Xue L. Ren X. Xu X. Chen C. (2024). Selective degradation of multimeric proteins by TRIM21-based molecular glue and PROTAC degraders. Cell 187 (25), 7126–7142.e20. 10.1016/j.cell.2024.10.015 39488207

[B34] Lu X. Kong X. Wu H. Hao J. Li S. Gu Z. (2023). UBE2M-mediated neddylation of TRIM21 regulates obesity-induced inflammation and metabolic disorders. Cell Metab. 35 (8), 1390–1405.e8. 10.1016/j.cmet.2023.05.011 37343564

[B35] MacDonald B. T. Tamai K. He X. (2009). Wnt/Beta-Catenin signaling: components, mechanisms, and diseases. Dev. Cell 17 (1), 9–26. 10.1016/j.devcel.2009.06.016 19619488 PMC2861485

[B36] Menendez J. A. Lupu R. (2007). Fatty acid synthase and the lipogenic phenotype in cancer pathogenesis. Nat. Rev. Cancer 7 (10), 763–777. 10.1038/nrc2222 17882277

[B37] Miller D. R. Thorburn A. (2021). Autophagy and organelle homeostasis in cancer. Dev. Cell 56 (7), 906–918. 10.1016/j.devcel.2021.02.010 33689692 PMC8026727

[B38] Mizushima N. Komatsu M. (2011). Autophagy: renovation of cells and tissues. Cell 147 (4), 728–741. 10.1016/j.cell.2011.10.026 22078875

[B39] Mo J. Su C. Li P. Yang Z. Tao R. Liu Q. (2025). CKAP4 in hepatocellular carcinoma: competitive RETREG1/FAM134B binding, reticulophagy regulation, and cancer progression. Autophagy 21 (4), 840–859. 10.1080/15548627.2024.2435236 39689859 PMC11925109

[B40] Paul S. Ghosh S. Kumar S. (2022). Tumor glycolysis, an essential sweet tooth of tumor cells. Semin. Cancer Biol. 86 (Pt 3), 1216–1230. 10.1016/j.semcancer.2022.09.007 36330953

[B41] Qian X. Cai J. Zhang Y. Shen S. Wang M. Liu S. (2024). EPDR1 promotes PD-L1 expression and tumor immune evasion by inhibiting TRIM21-dependent ubiquitylation of IkappaB kinase-β. EMBO J. 43 (19), 4248–4273. 10.1038/s44318-024-00201-6 39152265 PMC11445549

[B42] Semenza G. L. (2003). Targeting HIF-1 for cancer therapy. Nat. Rev. Cancer 3 (10), 721–732. 10.1038/nrc1187 13130303

[B43] Shi J. Zhang Z. Chen H. Y. Yao Y. Ke S. Yu K. (2025). Targeting the TRIM21-PD-1 axis potentiates immune checkpoint blockade and CAR-T cell therapy. Mol. Ther. 33 (3), 1073–1090. 10.1016/j.ymthe.2025.01.047 39905727 PMC11897759

[B44] TeSlaa T. Ralser M. Fan J. Rabinowitz J. D. (2023). The pentose phosphate pathway in health and disease. Nat. Metab. 5 (8), 1275–1289. 10.1038/s42255-023-00863-2 37612403 PMC11251397

[B45] Vinay D. S. Ryan E. P. Pawelec G. Talib W. H. Stagg J. Elkord E. (2015). Immune evasion in cancer: mechanistic basis and therapeutic strategies. Semin. Cancer Biol. 35 (Suppl. l), S185–S198. 10.1016/j.semcancer.2015.03.004 25818339

[B46] Wang L. Li H. Huang A. Zhao Y. Xiao C. Dong J. (2023). Mutual regulation between TRIM21 and TRIM8 *via* K48-linked ubiquitination. Oncogene 42 (50), 3708–3718. 10.1038/s41388-023-02879-0 37914816

[B57] Xiao K. Peng S. Lu J. Zhou T. Hong X. Chen S. (2023). UBE2S interacting with TRIM21 mediates the K11-linked ubiquitination of LPP to promote the lymphatic metastasis of bladder cancer. Cell Death Dis. 14 (07), 408. 10.1038/s41419-023-05938-2 37422473 PMC10329682

[B47] Yang K. Shi H. X. Liu X. Y. Shan Y. F. Wei B. Chen S. (2009). TRIM21 is essential to sustain IFN regulatory factor 3 activation during antiviral response. J. Immunol. 182 (6), 3782–3792. 10.4049/jimmunol.0803126 19265157

[B48] Yang P. Gao S. Shen J. Liu T. Lu K. Han X. (2025). TRIM21-mediated ubiquitination of SQSTM1/p62 abolishes its Ser403 phosphorylation and enhances palmitic acid cytotoxicity. Autophagy 21 (1), 178–190. 10.1080/15548627.2024.2394308 39172027 PMC11702951

[B49] Ye W. L. Huang L. Yang X. Q. Wan S. Gan W. J. Yang Y. (2024). TRIM21 induces selective autophagic degradation of c-Myc and sensitizes regorafenib therapy in colorectal cancer. Proc. Natl. Acad. Sci. U. S. A. 121 (42), e2406936121. 10.1073/pnas.2406936121 39388269 PMC11494295

[B50] Zahavi D. Hodge J. W. (2023). Targeting immunosuppressive adenosine signaling: a review of potential immunotherapy combination strategies. Int. J. Mol. Sci. 24 (10), 8871. 10.3390/ijms24108871 37240219 PMC10218801

[B51] Zanconato F. Cordenonsi M. Piccolo S. (2016). YAP/TAZ at the roots of cancer. Cancer Cell 29 (6), 783–803. 10.1016/j.ccell.2016.05.005 27300434 PMC6186419

[B52] Zhang M. Wang Z. Zhao Q. Yang Q. Bai J. Yang C. (2024). USP20 deubiquitinates and stabilizes the reticulophagy receptor RETREG1/FAM134B to drive reticulophagy. Autophagy 20 (8), 1780–1797. 10.1080/15548627.2024.2347103 38705724 PMC11262213

[B53] Zhang R. Shen Y. Zhang Q. Feng X. Liu X. Huo X. (2023). TRIM21-mediated Sohlh2 ubiquitination suppresses M2 macrophage polarization and progression of triple-negative breast cancer. Cell Death Dis. 14 (12), 850. 10.1038/s41419-023-06383-x 38123542 PMC10733312

[B54] Zhao L. Zhao J. Zhong K. Tong A. Jia D. (2022a). Targeted protein degradation: mechanisms, strategies and application. Signal Transduct. Target Ther. 7 (1), 113. 10.1038/s41392-022-00966-4 35379777 PMC8977435

[B55] Zhao R. He B. Bie Q. Cao J. Lu H. Zhang Z. (2022b). AQP5 complements LGR5 to determine the fates of gastric cancer stem cells through regulating ULK1 ubiquitination. J. Exp. Clin. Cancer Res. 41 (1), 322. 10.1186/s13046-022-02532-w 36372898 PMC9661769

[B56] Zhu Y. Banerjee A. Xie P. Ivanov A. A. Uddin A. Jiao Q. (2024). Pharmacological suppression of the OTUD4/CD73 proteolytic axis revives antitumor immunity against immune-suppressive breast cancers. J. Clin. Invest 134 (10), e176390. 10.1172/JCI176390 38530357 PMC11093616

